# The complete mitochondrial genome of *Atheraster arandae* (Asteroidea: Valvatida: Goniasteridae) from the western Pacific

**DOI:** 10.1080/23802359.2026.2620182

**Published:** 2026-02-14

**Authors:** Deming Kong, Ning Xiao, Xinzheng Li, Lixing Wang, Tianyue Peng

**Affiliations:** a Guangzhou Marine Geological Survey, China Geological Survey, Guangzhou, China; b Department of Marine Organism Taxonomy and Phylogeny, Institute of Oceanology, Chinese Academy of Sciences, Qingdao, China; c University of Chinese Academy of Sciences, Beijing, China; d Marine Biological Museum, Chinese Academy of Sciences, China

**Keywords:** *Atheraster arandae*, deep-sea, mitogenome, Valvatida, phylogeny

## Abstract

This study presents the complete mitogenome sequence of *Atheraster arandae*, providing valuable information for its genetic and taxonomic research. Through next-generation sequencing, we successfully obtained the complete mitogenome of *A. arandae*, with a length of 16,292 bp. The mitogenome consisted of 13 protein-coding genes (PCGs), 22 transfer RNA (tRNA) genes, and 2 ribosomal RNA (rRNA) genes. Both Maximum Likelihood and Bayesian inference phylogenetic trees demonstrated that *A. arandae* clustered closely with *Iconaster longimanus*. By revealing its mitogenome and positioning its phylogenetic placement within the Goniasteridae lineage, this study provides insights into the phylogenetic context of *A. arandae*.

## Introduction

*Atheraster arandae* (Mah 2006) belongs to the family Goniasteridae within the order Valvatida, class Asteroidea. This species was originally presented as *Circeaster arandae* by Mah (Mah 2006) and subsequently transferred to the genus *Atheraster* (Mah 2022). Known from localities in the Madagascar, New Caledonia, and Hawaiian Islands, *A. arandae* exhibits a broad distribution in the deep seas from the Indian Ocean to the North Pacific (Mah 2006, 2022, 2024). As the most diverse asteroid order, Valvatida harbors unresolved phylogenetic issues, including questions about its monophyletic status and interfamilial relationships (Mah and Blake 2012; Linchangco et al. 2017; Sun et al. 2022).

The mitogenome, a circular genome present in eukaryotic cells, has become an important molecular marker for phylogenetic studies in echinoderms. By comparing sequence variations, gene arrangement patterns, nucleotide composition biases, and selection pressures in mitogenomes, it is possible to effectively resolve evolutionary relationships among various echinoderm groups and even reveal cryptic species diversity (Xu et al. 2024; Mu et al. 2025). By reconstructing a maximum likelihood and phylogenetic inference tree through the measurement of the complete mitogenome of *Linckia laevigata* and integration with 25 published asteroidean mitogenomes, Shimpei F. Hiruta’s study confirmed the paraphyly of Valvatida and revealed the unstable phylogenetic positions of Ophidiasteridae and Goniasteridae (Hiruta et al. 2020). Nina Yasuda estimated the intraspecific divergence time of *Acanthaster planci* using a concatenated dataset of 13 PCGs, demonstrating that mitogenomic data offer high phylogenetic resolution at the population level (Yasuda et al. 2006). We report the first complete mitogenome for *A. arandae*, containing the standard metazoan complement of 13 PCGs, 22 tRNA genes, and 2 rRNA genes. This novel genomic resource is crucial for clarifying the phylogenetic position of Goniasteridae, shedding light on their evolutionary diversification, taxonomy, and biogeographic history within the Asteroidea.

## Materials and methods

**Figure 1.**
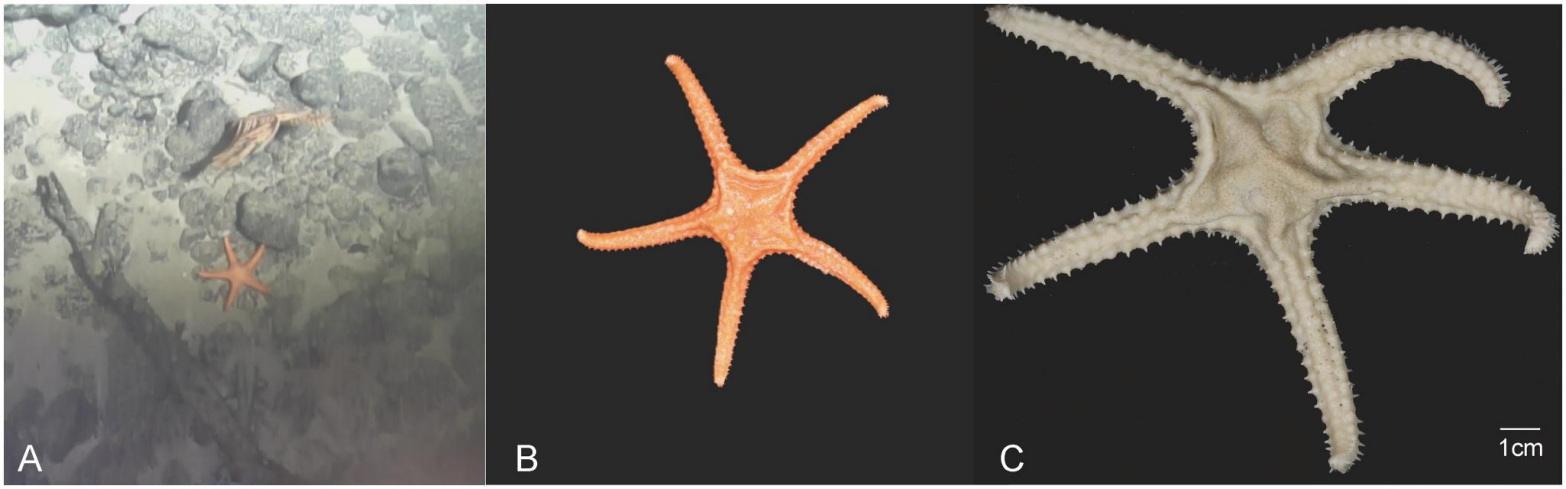
Photograph of *Atheraster arandae* (Mah, 2006) collected from the western Pacific: A. In-situ photograph; B. fresh specimen; C. Alcohol sample. The photographs were taken by Jixing Sui.

*Atheraster arandae* was collected from a seamount in the western Pacific (16°36′ N, 134°43′ E, depth 2,140 m) in July 2024 by the remotely operated vehicle ‘Haima’ and preserved immediately frozen in −80 °C (Figure 1). The sample has been deposited at the Guangzhou Marine Geological Survey, China Geological Survey, Guangzhou, China, with the voucher number: GMGS-AS-S01-HSL2024PH (contact: Tianyue Peng, E-mail: pengtianyue@mail.cgs.gov.cn). Genomic DNA isolation from gonadal tissue was performed using the DNeasy Tissue Kit (Qiagen, Beijing, China) following the manufacturer’s protocols. After extracting, 1 μg of purified DNA was fragmented to approximately 500 bp using the Covaris M220 system (Covaris LLC., Woburn, Massachusetts, USA), following the manufacturer’s instructions. These fragmented DNA samples (∼500 bp) were subsequently used for the construction of short-insert libraries (TruSeq™ Nano DNA Sample Prep Kit, Illumina), and then were sequenced on the Illumina NovaSeq 6000 platform (BIOZERON Co., Ltd, Shanghai, China), generating 150 bp paired-end reads length (SAMN51337969).

The mitogenome was reconstructed through a hybrid approach that combined de novo assembly and reference-guided assembly (Kurtz et al. 2004; Camacho et al. 2009; Meng et al. 2019). The mitochondrial gene annotation was performed using the online MITOS 2 (Bernt et al. 2013)and GeSeq (Tillich et al. 2017), and the default parameters were applied for the prediction of PCGs, tRNA genes, and rRNA genes. The position of each coding gene was determined using BLAST searches against reference mitogenome genes. Using the SnapGene Viewer (https://www.snapgene.com), manual correction of the start and stop codons of genes was performed, guided by the reference mitogenome. The circular mitogenome map was drawn using the CGview tool (Grant and Stothard 2008).

PhyloSuite is used for processing and phylogenic tree construction of mitogenomic data (Zhang et al. 2019a, 2019b). MAFFT 7.313 was used to align the nucleotide sequences of 13 PCGs, respectively (Katoh and Standley 2013), and Gblock V 0.91b was used to select for conserved sites in the sequence with default setting (Talavera and Castresana 2007). The aligned nucleotide sequences of PCGs were concatenated into a dataset. Subsequently, ModelFinder was applied to identify the optimal partitioning scheme for the dataset and determine the best nucleotide substitution model for each partition (Kalyaanamoorthy et al. 2017). The ML tree was reconstructed using IQ-TREE (Nguyen et al. 2015), with ultrafast bootstrap (BS) set to 5000. Bayesian trees were built using MrBayes 3.2.6 (Ronquist et al. 2012). The Markov chain Monte Carlo (MCMC) was set to run for 2 million generations, sampling every 1000 generations, and the first 25% of the trees were discarded (burn-in), the remaining trees were used to summarize the consensus tree and to estimate the posterior probabilities. Effective sample size (ESS) values for all parameters were checked by Tracer 1.7 (https://www.beast2.org/treeannotator/) to ensure convergence. Phylogenetic trees and gene sequences are graphically edited using iTOL (Letunic and Bork 2007).

## Result

**Figure 2.**
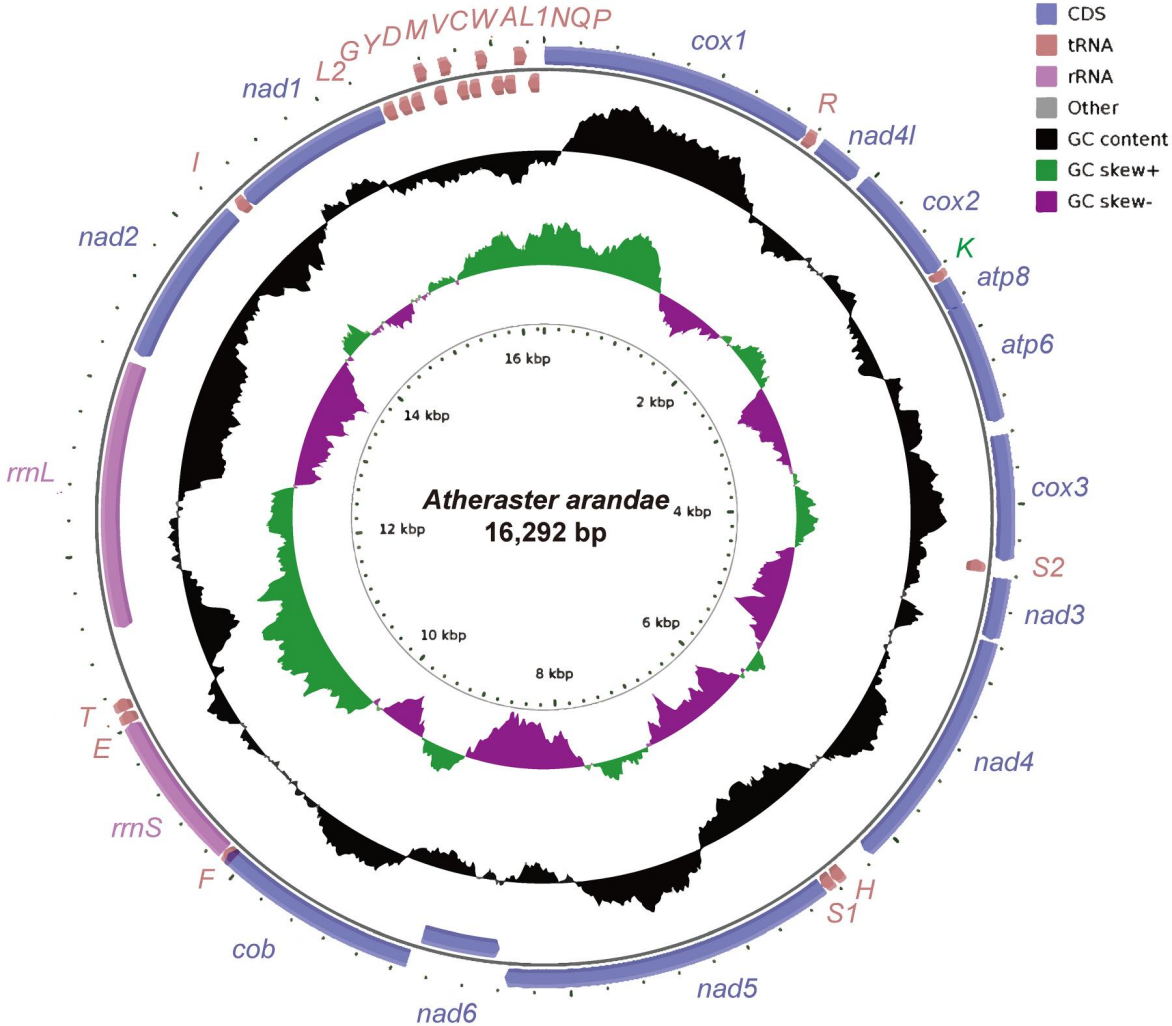
Circular map of the *Atheraster arandae* mitogenome. Different colors indicate different types of genes and regions.

The complete mitogenome of *A. arandae* is 16,292 bp in length (Figure 2) and the average coverage depth was 7935× (Figure S1). The mitogenome contains the typical metazoan complement of 13 PCGs, 22 tRNA genes, and 2 rRNA genes. Nucleotide composition analysis revealed a base distribution of A (34.8%), T (27.6%), G (11.4%), and C (26.2%), resulting in an overall A + T content of 62.4%, which is near the average for asteroid species (Mu et al. 2018). Among the PCGs, *ND1* and *ND5* utilize GTG as start codon, while *ND3* and *ND4L* initiate with ATT. All other PCGs begin with the ATG start codon.

**Figure 3.**
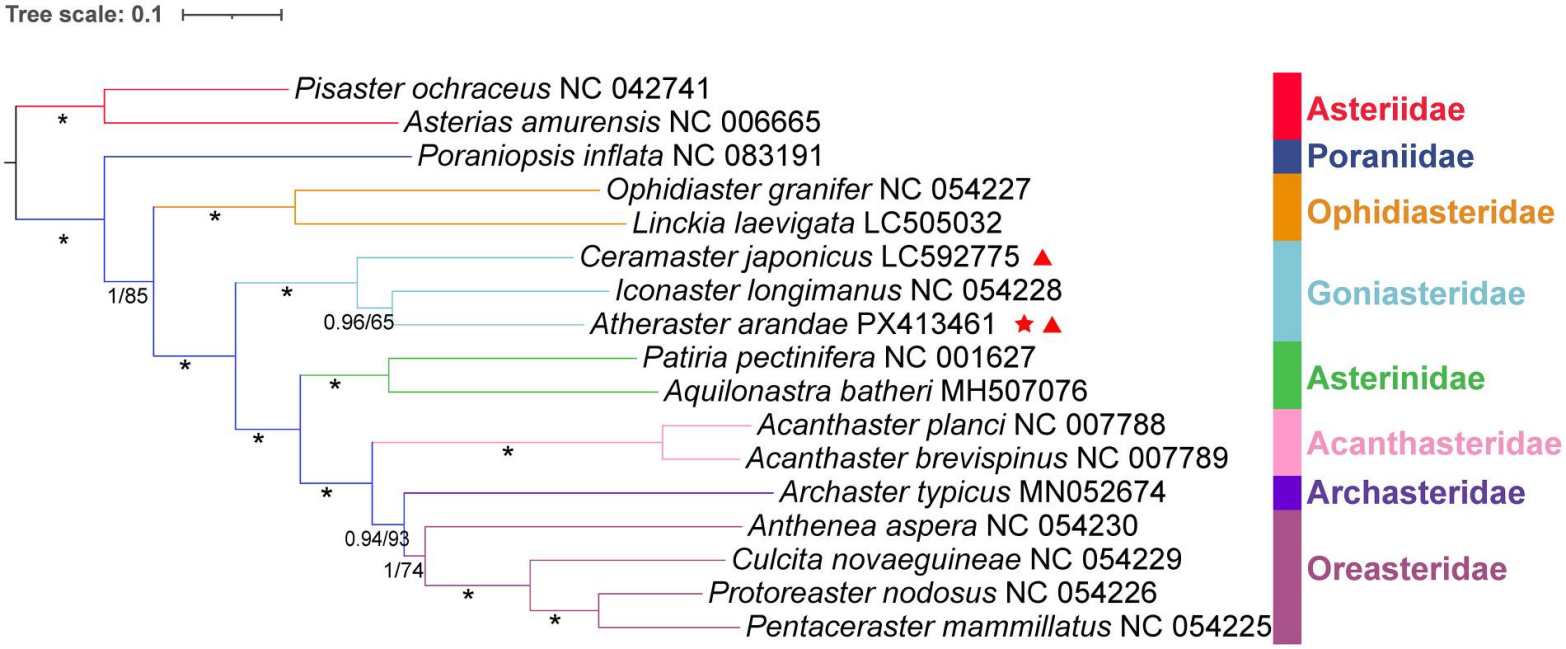
Maximum likelihood and Bayesian inference tree based on the concatenated nucleotide sequence of 13 protein-coding genes of *Atheraster arandae* and 16 asteroid species. Nodal values are ML bootstrap support values (BS) and BI Posterior Probability (PP). An asterisk (*) indicates 100% BS and 1.0 PP. Red asterisks indicate mitogenome data provided by this study. Red triangles denote deep-sea species. The following sequences were used (Table S1): Acanthaster brevispinus NC_007789 (Yasuda et al. 2006b), Acanthaster planci NC_007788 (Yasuda et al. 2006b), Anthenea aspera NC_054230 (Quek et al. 2021), Aquilonastra batheri MH507076 (Lee and Shin 2018), Archaster typicus MN052674 (Quek et al. 2019), Asterias amurensis NC_006665 (Matsubara et al. 2005), Atheraster arandae PX413461 (this study), Ceramaster japonicus LC592775 (Unpublished), Culcita novaeguineae NC_054229 (Quek et al. 2021), Iconaster longimanus NC_054228 (Quek et al. 2021), Linckia laevigata LC505032 (Hiruta et al. 2020), Ophidiaster granifer NC_054227 (Quek et al. 2021), Patiria miniata ON478348 (Thimmappa et al. 2022), Patiria pectinifera NC_001627 (Asakawa et al. 1995), Pentaceraster mammillatus NC_054225 (Quek et al. 2021), Pisaster ochraceus NC_042741 (Unpublished), Poraniopsis inflata NC_083191 (Alboasud et al. 2024), Protoreaster nodosus (Quek et al. 2021).

Phylogenetic trees reconstructed from the concatenated dataset of 13 PCGs using both Maximum Likelihood (ML) and Bayesian Inference (BI) methods consistently placed *A. arandae* within a strongly supported clade as sister to species belonging to the Goniasteridae family (*Iconaster longimanus* and *Ceramaster japonicus*) (Figure 3). The families Goniasteridae, Ophidiasteridae, Asterinidae, Acanthasteridae, and Oreasteridae were each recovered as monophyletic. Notably, the Ophidiasteridae occupied a more basal position relative to the Goniasteridae. Furthermore, the deep-sea starfish shares identical mitochondrial gene order with its shallow-water relatives (Figure S2).

## Discussion and conclusion

The mitogenome, as a relatively independent hereditary material outside the cell nucleus, exhibits distinctive traits such as the absence of genetic recombination, maternal inheritance, and higher mutation rate (Yamauchi et al. 2004; Elson and Lightowlers 2006; Qian et al. 2011). Furthermore, mitogenomes provide abundant and valuable evolutionary information at the levels of sequence and gene arrangement, which have been extensively employed in phylogenetic and evolutionary research across diverse metazoan taxa (Boore 1999; Hong-ying et al. 2003; Tan et al. 2019). Therefore, we present the first complete mitogenome of *A. arandae* in this study. Comparative analysis revealed that the basic composition of the mitogenome of *A. arandae* exhibits high similarity to those of most previously studied asteroids.

Previous molecular phylogenetic studies (Sun et al. 2022) indicate that Valvatida is not monophyletic but paraphyletic. The order is consistently divided into two primary clades. Furthermore, the phylogenetic placement of constituent families such as Ophidiasteridae and Goniasteridae exhibits significant variation depending on the analytical method employed (Sun et al. 2022). This study significantly advances our understanding of the phylogenetic relationships of *A. arandae* and provides valuable insights into the phylogeny within Valvatida.

Mitochondrial gene order has been demonstrated as an effective approach for resolving deep phylogenetic relationships (Boore 1999). This study compared mitochondrial gene orders among asteroidea animals, anticipating that starfish mt gene orders might reveal phylogenetic insights. However, the gene orders of eight asteroidea family species were identical, consistent with findings reported in previous studies (Mu et al. 2018). This study presents the first complete mitogenome of *Atheraster arandae*, enriching the deep-sea goniasterids genetic database and providing critical insights into its phylogeny. These findings advance our understanding of mitogenome evolution in deep-sea echinoderms and establish a novel framework for future studies on deep-sea organismal evolution.

## Supplementary Material

Supplemental Material

Supplemental Material

## Data Availability

The genome sequence data that support the findings of this study are openly available in GenBank of NCBI at [https://www.ncbi.nlm.nih.gov] (https://www.ncbi.nlm.nih.gov/) under the accession no. PX413461. The associated BioProject, SRA, and Bio-Sample numbers are PRJNA1328624, SAMN51337969, and SRR35427775 respectively.
