# Supplementary figures and images for: The complete mitochondrial genome of Atheraster arandae (Asteroidea: Valvatida: Goniasteridae) from the western Pacific
Source: Mitochondrial DNA B Resour. 2026 Feb 14;11(3):409–13. doi: 10.1080/23802359.2026.2620182 (PMC12912233; doi:10.1080/23802359.2026.2620182)

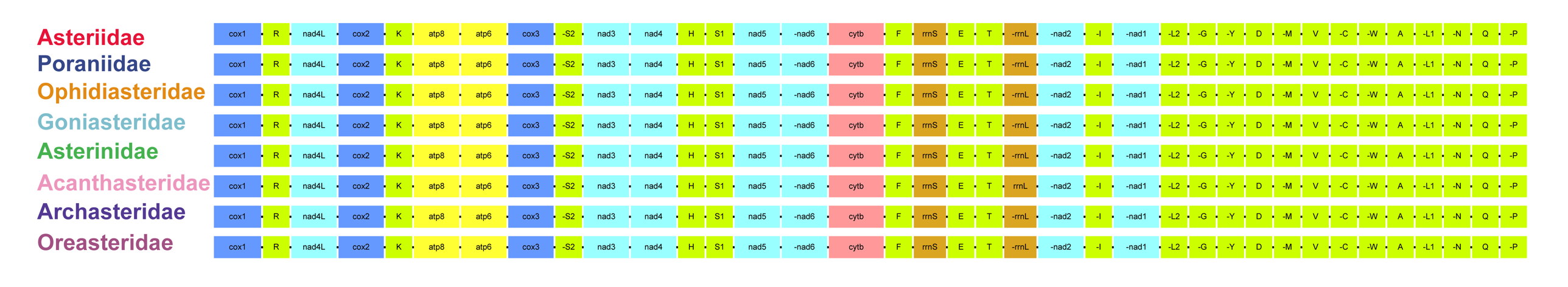

Supplement: Supplemental Material [file TMDN_A_2620182_SM5563.tif]

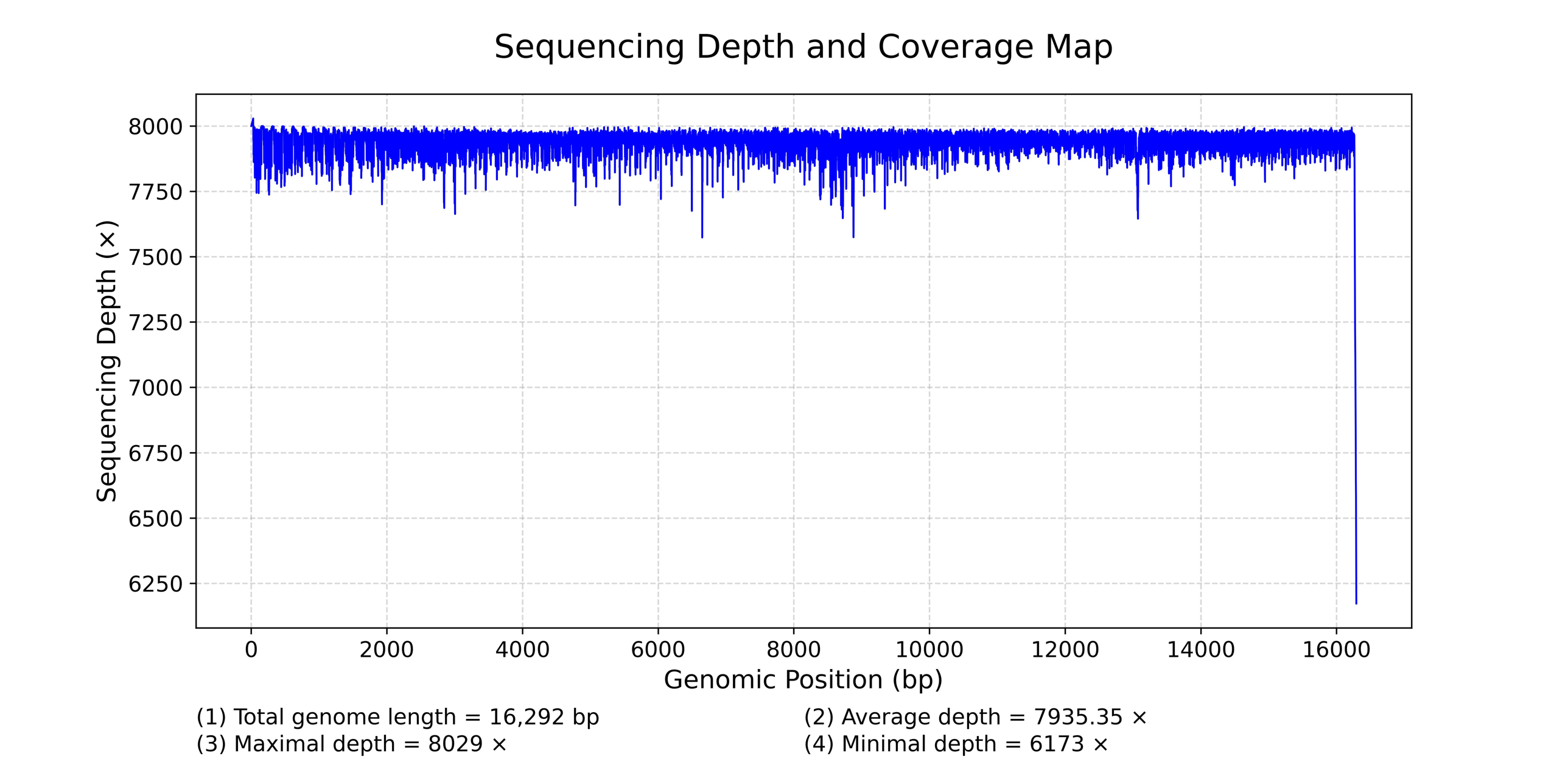

Supplement: Supplemental Material [file TMDN_A_2620182_SM5562.tif]
